# Comparative Transcriptome Analysis in Monocyte-Derived Macrophages of Asymptomatic *GBA* Mutation Carriers and Patients with GBA-Associated Parkinson’s Disease

**DOI:** 10.3390/genes12101545

**Published:** 2021-09-29

**Authors:** Tatiana Usenko, Anastasia Bezrukova, Katerina Basharova, Alexandra Panteleeva, Mikhail Nikolaev, Alena Kopytova, Irina Miliukhina, Anton Emelyanov, Ekaterina Zakharova, Sofya Pchelina

**Affiliations:** 1 Petersburg Nuclear Physics Institute Named by B.P. Konstantinov of National Research Centre «Kurchatov Institute», Gatchina 188300, Russia; bezrukova_ai@pnpi.nrcki.ru (A.B.); kbasharova@yandex.ru (K.B.); panteleeva_aa@pnpi.nrcki.ru (A.P.); nikolaev_ma@pnpi.nrcki.ru (M.N.); kopytova_ae@pnpi.nrcki.ru (A.K.); milyukhinaiv@yandex.ru (I.M.); emelyanov_ak@pnpi.nrcki.ru (A.E.); pchelina_sn@pnpi.nrcki.ru (S.P.); 2 Pavlov First Saint-Petersburg State Medical University, Saint-Petersburg 197022, Russia; 3 Institute of the Human Brain of RAS, Saint-Petersburg 197376, Russia; 4 Research Center for Medical Genetics, Moscow 115522, Russia; doctor.zakharova@gmail.com

**Keywords:** Parkinson’s disease, *GBA*, macrophages, RNA-seq, transcriptome

## Abstract

Mutations of the *GBA* gene, encoding for lysosomal enzyme glucocerebrosidase (GCase), are the greatest genetic risk factor for Parkinson’s disease (PD) with frequency between 5% and 20% across the world. N370S and L444P are the two most common mutations in the *GBA* gene. PD carriers of severe mutation L444P in the *GBA* gene is characterized by the earlier age at onset compared to N370S. Not every carrier of *GBA* mutations develop PD during one’s lifetime. In the current study we aimed to find common gene expression signatures in PD associated with mutation in the *GBA* gene (GBA-PD) using RNA-seq. We compared transcriptome of monocyte-derived macrophages of 5 patients with GBA-PD (4 L444P/N, 1 N370S/N) and 4 asymptomatic *GBA* mutation carriers (GBA-carriers) (3 L444P/N, 1 N370S/N) and 4 controls. We also conducted comparative transcriptome analysis for L444P/N only GBA-PD patients and GBA-carriers. Revealed deregulated genes in GBA-PD independently of *GBA* mutations (L444P or N370S) were involved in immune response, neuronal function. We found upregulated pathway associated with zinc metabolism in L444P/N GBA-PD patients. The potential important role of *DUSP1* in the pathogenesis of GBA-PD was suggested.

## 1. Introduction

Parkinson’s disease (PD) is a neurodegenerative disorder that is characterized by the accumulation of abnormal protein aggregates of alpha-synuclein in the brain [1,2]. Several genetic factors have been associated with an increased risk of PD development. Mutations in the *GBA* gene are the highest genetic risk factors for PD with an increase of PD risk (of seven to eight times) and with a frequency of 5% to 20% in all populations [3,4]. The *GBA* gene, encoding the lysosomal enzyme glucocerebrosidase (GCase), is the key enzyme in ceramide metabolism and catalyzes the hydrolysis of glucosylceramide to glucose and ceramide. GCase is expressed in most tissues, especially in the brain, endocrine issue, liver, spleen, skin (https://www.proteinatlas.org, accessed on 21 September 2021). *GBA* mutations resulted in the most common lysosomal storage disorder (LSD), Gaucher disease (GD), characterized with lysosphingolipid accumulation, presumably in blood macrophages. Generally, the two most common mutations in the *GBA* gene N370S (c.1226A > G) and L444P (c.1448 T > C) account for 60–70% of the mutant alleles amongst others [4,5]. PD carriers of the severe L444P mutation in the *GBA* gene are characterized by an earlier age at onset and rapid progression [6] compared to N370S and other mild mutations. The molecular mechanisms of an association between *GBA* mutations and PD are unclear [7]. We, and others, have previously demonstrated that mutations in the *GBA* gene lead to a decrease of GCase activity and an increase of blood lysosphingolipid concentration, even in heterozygous carriers of *GBA* mutations [8,9,10,11]. However, not all carriers of *GBA* mutations develop PD. GCase dysfunction does not seem to be enough to launch the pathogenic mechanism of PD among *GBA* mutation carriers. 

Transcriptome analysis using next-generation sequencing (RNA-seq) is a powerful method to analyze the genome transcriptomic profile with high-resolution. Although variations in the transcriptome are tissue specific, the blood and brain demonstrated significant gene expression similarities [12,13]. It is worth noting that RNA-seq revealed the difference between transcriptomic profiles in the peripheral blood of symptomatic and asymptomatic G2019S *LRRK2* mutation carriers and identified common differentially expression genes functionally involved in the pathways and related with LRRK2-PD pathogenesis, such as Akt signaling, glucose metabolism, or immunity [14,15]. Monocyte-derived macrophages represent one of the most promising models for investigating molecular mechanisms of GCase dysfunction, as this cell type is vulnerable for disturbances in ceramide metabolism [16,17]. In particular, we and others demonstrated high potential of peripheral blood monocyte-derived macrophages to reflect individual sensitivity for drugs influencing GCase activity [18,19]. Here, we first generated the transcriptomic profiles for GBA-PD patients, asymptomatic *GBA* mutation carriers (GBA carriers), and controls in monocyte-derived macrophages, in order to investigate what variations in monocyte-derived macrophage transcriptomes can be attributed to the presence of *GBA* mutation and what can be viewed as a trigger of PD in *GBA* mutation carriers. Our results will be useful to others looking for potential triggers of PD among *GBA* mutation carriers, and provides future directions for PD preclinical research.

## 2. Materials and Methods

This project was approved by the Pavlov First Saint-Petersburg State Medical University. A formal written consent form was provided to all included subjects to read and sign prior to the study. 

### 2.1. Subjects

Five patients with GBA-PD, four GBA carriers, and four neurologically healthy individuals were enrolled for the current study. Demographic data of the studied groups are summarized in Table 1. Controls had no history of parkinsonism. GBA-PD patients were diagnosed at two neurological clinic centers in St. Petersburg, Russia: Pavlov First Saint-Petersburg State Medical University and the Institute of the Human Brain of RAS. A standard neurologic clinical examination was performed for all participants and the diagnosis of PD was based on previously published criteria [20]. GBA-PD patients were recruited by genotyping of N370S, L444P mutations in the *GBA* gene among PD patients, as previously described [3]. GBA carriers were collected from first-degree relatives of GD patients at the Research Centre for Medical Genetics where *GBA* mutations were confirmed by target sequencing of all exons in the *GBA* gene.

### 2.2. Differentiation of Human Monocytes to Macrophages

Peripheral blood mononuclear cells (PBMCs) were isolated from 24 mL of peripheral blood from participants, by density gradient centrifugation (Ficoll–Paque PLUS, GE Healthcare, Chicago, IL, USA). PBMCs were differentiated by the macrophage colony-stimulating factor (M-CSF) (10 ng/ml) (Sigma-Aldrich, Burlington, MA, USA) in RPMI 1640 medium (Gibco, Waltham, MA, USA) supplemented with 10% FCS (Gibco, Waltham, MA, USA) with harvesting after 5 days. Phenotypical maturation of monocyte-derived macrophages was confirmed by light microscopy and flow cytometry with specific antibodies to CD14+ and CD68+ (eBioscience, San Diego, CA, USA), as described earlier [18,21].

### 2.3. RNA Isolation and RNA Sequencing (RNA-Seq)

RNA was isolated from monocyte-derived macrophages and amplified following the user manual of the SMART-Seq™ v4 Ultra™ Low Input RNA Kit for sequencing. Sequencing libraries were generated using the NEBNext^®^ Ultra™ DNA Library Prep Kit for Illumina^®^ (NEB, Ipswich, MA, USA), following the manufacturer’s recommendations. The RNA molecules that contained polyA were then sequenced on the Illumina HiSeq1500 platform.

### 2.4. Quality Control

Quality control for each sample was performed by FastQC (v0.11.9) [22] and RSeQC (v4.0.0)) [23]. In this step, clean data (clean reads) were obtained by removing low-quality reads, reads containing adapters, and reads containing ploy-N from raw data. The removal adapter was conducted with Cutadapt [24]. All downstream analyses were based on clean data. 

### 2.5. Reads Mapping to Reference Genome

Human reference genome assembly GRCh38 (hg38) and gene model annotation files were downloaded from the Gencode website (https://www.gencodegenes.org/human/ (accessed on 9 September 2021)) directly (release 37). HISAT2 (v2.2.1) [25] was used with default parameters to build the index of the reference genome and mapping reads to the genome.

### 2.6. Quantification of Gene Expression Level 

Counting sequencing reads mapping to each gene after the alignment step was performed using the HTSeq-count function from the HTSeq framework (v.0.6.1) [26]. 

### 2.7. Analysis of Gene Differential Expression

Gene differential expression analyses of three groups were performed using the DESeq2 package (v.1.30.1) [27] in R (v.4.0.3). DESeq2 provides statistical routines for determining differential expression in digital gene expression data using a model based on negative binomial distribution. The resulting *p*-values were adjusted using Benjamini and Hochberg’s approach for controlling the false discovery rate (FDR). Detected differential expression of genes was considered statistically significant at FDR ≤ 0.05 and a fold change (FC) threshold >1.5. The differentially expressed genes were visualized in a volcano plot built by using ggplot (v.3.3.3) in R (v4.0.3). 

### 2.8. GO Enrichment Analysis of Differentially Expressed Genes 

Gene Ontology (GO) enrichment analysis of differentially expressed genes was performed using GO resource (http://geneontology.org (accessed on 9 September 2021)) and was carried out using the apps ClueGO v. 2.5.7 [28] and CluePedia v. 1.5.3 [29] for Cytoscape v. 3.6.1. GO terms with a corrected *p*-value of less than 0.05. Term groups were selected by ClueGO based on the number of common genes/terms (>50%). Term clusters were selected based on common genes. A network of selected metabolic processes and DEGs was built using CluePedia v. 1.5.7. 

## 3. Results

### 3.1. RNA-Seq Experiments

A whole-transcriptome analysis of monocyte-derived macrophages obtained from four patients with L444P/N GBA-PD, three L444P/N GBA carriers, and controls without any *GBA* mutations (*N* = 4) was performed. Transcriptome analysis of monocyte-derived macrophages was also conducted for all GBA-PD patients (L444P/N, *N* = 4, N370S/N, *N* = 1), and GBA carriers (L444P/N, *N* = 3, N370S/N, *N* = 1). Using the Illumina HiSeq 1500 sequencer, we generated 10–14M raw reads, trimming from the 13 samples, with a read length of 50 bp. After strict quality control, more than 20G clean bases were retained. Overall, 21,980 genes were identified in each of the 13 samples. Post-trimming and mapping results for all groups are provided in Table S1. Between 85.20% and 95.97% of the clean reads was aligned to the reference genome. Raw data were subjected to differential expression testing with DESeq2.

### 3.2. Changes in the Transcriptome Attributed to the Presence of GBA L444P/N Mutation 

**Figure 1:**
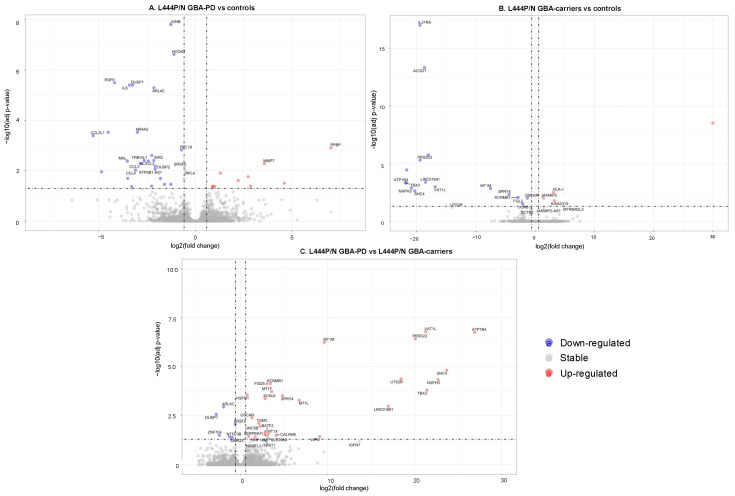
Volcano plot for DEGs between the studied groups (FDR < 0.05 and |FC| > 1.5); the upregulated genes are represented by red dots and the downregulated genes are represented by blue dots. (**A**). L444P/N GBA-PD patients and controls, (**B**). L444P/N GBA carriers and controls, (**C**). L444P/N GBA-PD patients, and L444P/N GBA carriers. (GBA-PD—Parkinson’s disease associated with mutations in the *GBA* gene; GBA carriers—asymptomatic *GBA* mutation carriers; DEGs—differentially expressed genes).

**Figure 2:**
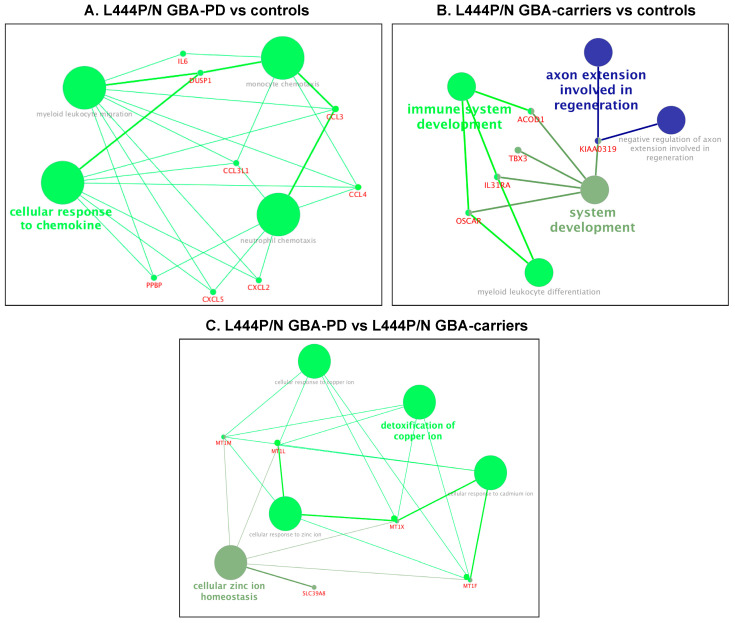
Networks of selected metabolic processes and DEGs in (**A**). L444P/N GBA-PD vs. controls; (**B**). L444P/N GBA carriers vs. controls; (**C**). L444P/N GBA-PD vs. L444P/N GBA carriers (obtained using CluePedia v. 1.5.7 + ClueGo v.2.5.7). (GBA-PD—Parkinson’s disease associated with mutations in the *GBA* gene; GBA carriers—asymptomatic *GBA* mutation carriers; DEGs—differentially expressed genes).

**Figure 3:**
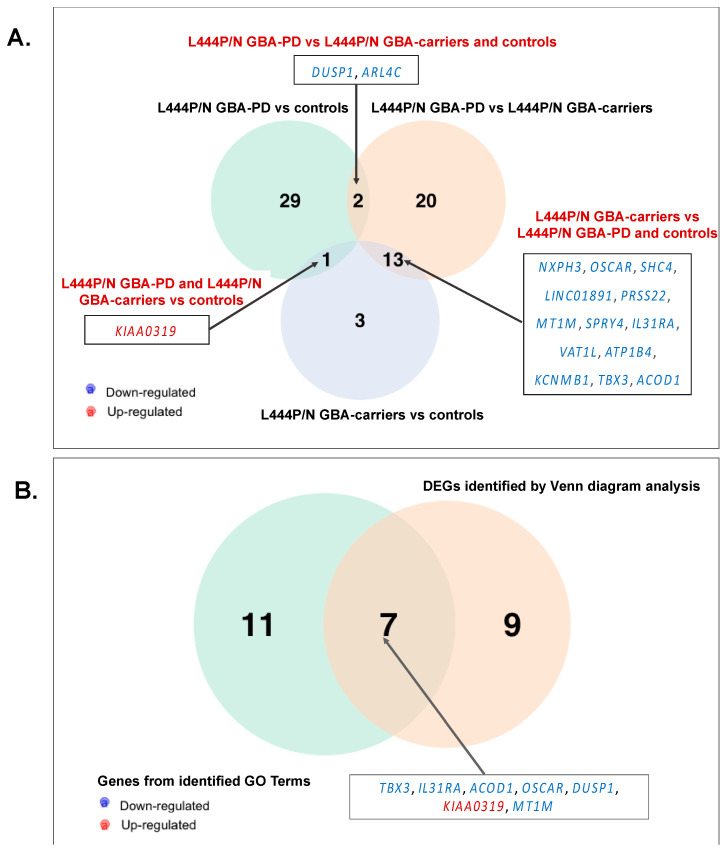
Venn diagram of (**A**). DEGs in monocyte-derived macrophages of L444P/N GBA-PD patients to controls compared to L444P/N GBA-PD patients to L444P/N GBA carriers, and compared to L444P/N GBA carriers and controls. B. DEGs determined be the Venn diagram in (**B**) and DEGs determined by GO analysis for L444P/N GBA-PD patients, L444P/N GBA carriers, controls. All data are presented as the number of genes with a *p*-value < 0.05 and |FC| more than 1.5. Three Venn diagrams were developed using the library VennDiagram (v.1.6.20) in R (v.4.0.3). (GBA-PD—Parkinson’s disease associated with mutations in the *GBA* gene; GBA carriers—asymptomatic *GBA* mutation carriers; DEGs—differentially expressed genes; GO—gene ontology).

First, we conducted comparative transcriptome analysis of GBA-PD patients baring L444P/N mutation and controls, which revealed 32 DEGs, and asymptomatic carriers of the *GBA* L444P/N mutation and controls, which revealed 18 DEGs (Tables S2 and S3, Figure 1A,B). Moreover, 36 DEGs were revealed between L444P/N GBA-PD patients and L444P/N GBA carriers (Table S4, Figure 1C). The top list of revealed DEGs in L444P/N GBA-PD patients compared to controls included the genes, *JUNB, NR4A2,* and *EGR1*, which played roles in neurogenesis. GBA-PD was characterized by downregulated expression of those genes. GO term enrichment analysis was conducted for all determined DEGs. We considered “metabolic process” terms with a *p*-value (Bonferroni corrected) <0.05 and all types of GO terms to gene connections. Significant terms are presented in Table 2 and networks are performed (Figure 2A–C). Pathways from GO databases enriched by DEGs that were found when comparing GBA-PD patients to the controls were associated with cytokine secretion (cellular response to chemokine (GO:1990869) and immune response (monocyte chemotaxis (GO:0002548), neutrophil chemotaxis (GO:0030593), and myeloid leukocyte migration (GO: 0097529)) (Table 2, Figure 2A). Altered biological GO pathways in L444P/N GBA-PD patients compared to L444P/N GBA carriers were the pathways related to cellular response to cadmium ion (GO:0071276), cellular response to zinc ion (GO:0071294), cellular zinc ion homeostasis (GO:0006882), detoxification of copper ion (GO:0010273), cellular response to copper ion (GO:0071280) (Table 2, Figure 2B). The 13 genes deregulated in L444P/N GBA carriers compared to the controls were involved in the enriched pathways related to immune response (system development (GO:0048731), immune system development (GO:0002520), myeloid leukocytes differentiation (GO:0002573)), and regulation negation axon extension involved in regeneration (GO:0048692) and axon extension involved in regeneration (GO:0048677) (Table 2, Figure 2C). The Venn diagram demonstrated one upregulated DEG, *KIAA0319*, which was upregulated in both L444P/N GBA-PD patients and L444P/N GBA carriers compared to the controls and two DEGs, *DUSP1* and *ARL4C*, which were downregulated in L444P/N GBA-PD patients compared to both L444P/N GBA carriers and controls (Figure 3A). The comparison between the list of DEGs from the GO analysis and the list of DEGs obtained by the Venn diagram revealed five genes (*IL31RA*, *ACOD1*, *OSCAR*, *MT1M*, *TBX3*) downregulated in L444P/N GBA carriers compared to L444P/N GBA-PD and controls, and one downregulated gene (*DUSP1*) in L444P/N GBA-PD patients compared to L444P/N GBA carriers and controls (Figure 3A), and one upregulated gene (*KIAA0319*) in L444P/N GBA-PD patients and L444P/N GBA carriers compared to controls (Figure 3B).

### 3.3. Differentially Expressed Genes and Enriched Pathways in GBA-PD Patients (L444P/N +N370S/N) and GBA Carriers (L444P/N +N370S/N) Compared to Controls

**Figure 4:**
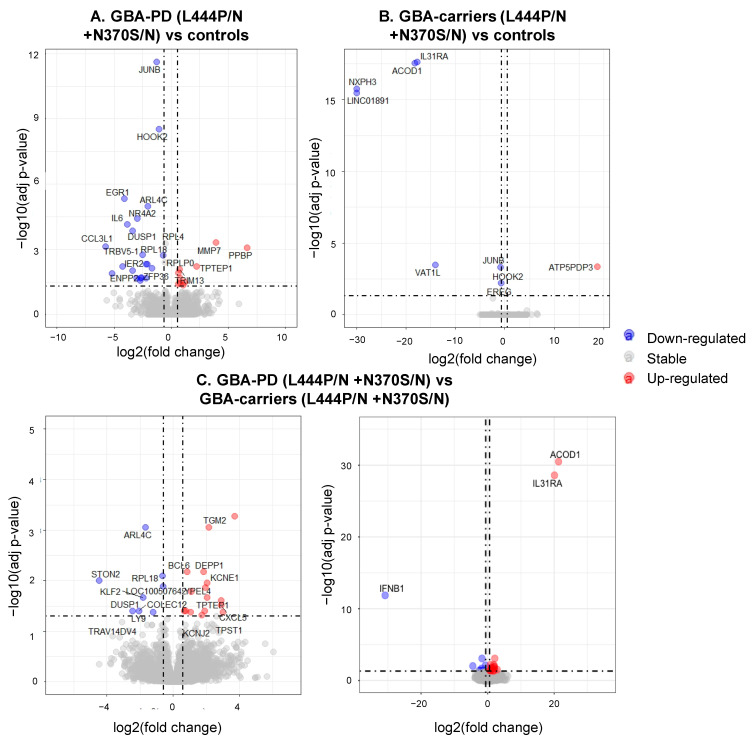
Volcano plot for DEGs between the studied groups (FDR < 0.05 and |FC| > 1.5); the upregulated genes are represented by red dots and the downregulated genes are represented by blue dots. (**A**). GBA-PD patients and controls; (**B**). GBA carriers and controls; (**C**). GBA-PD patients and GBA carriers. (GBA-PD—Parkinson’s disease associated with mutations in the *GBA* gene; GBA carriers—asymptomatic *GBA* mutation carriers; DEGs—differentially expressed genes).

**Figure 5:**
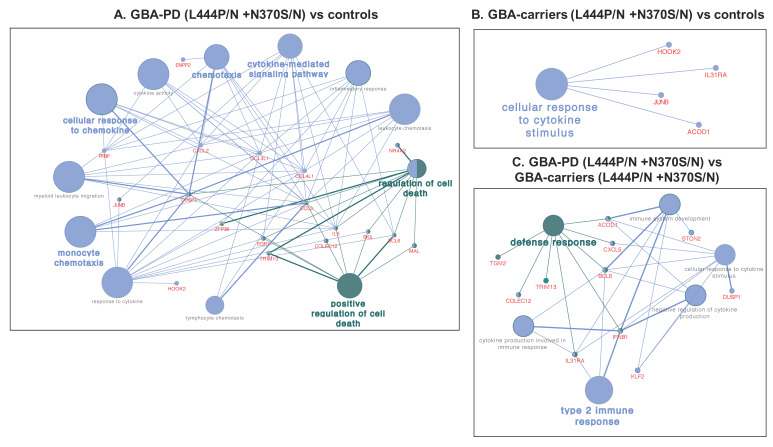
Networks of selected metabolic processes and DEGs in (**A**). GBA-PD (L444P/N +N370S/N) vs. controls; (**B**). GBA carriers (L444P/N +N370S/N) vs. controls; (**C**). GBA-PD (L444P/N +N370S/N) vs. GBA carriers (L444P/N +N370S/N) (obtained using CluePedia v. 1.5.7 + ClueGo v.2.5.7). (GBA-PD—Parkinson’s disease associated with mutations in the *GBA* gene; GBA carriers—asymptomatic *GBA* mutation carriers; DEGs—differentially expressed genes).

**Figure 6:**
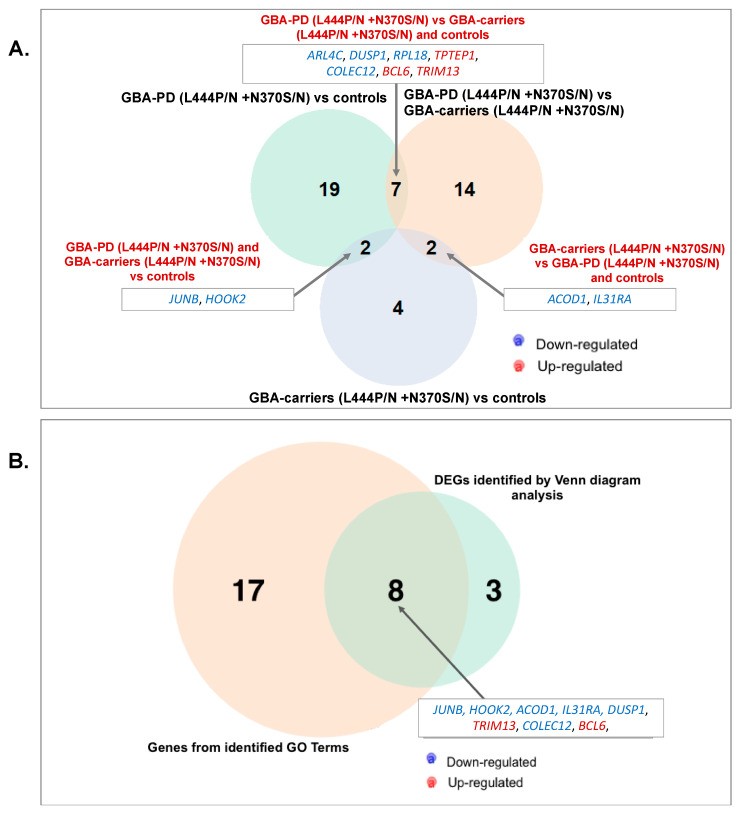
Venn diagram of (**A**). DEGs in monocyte-derived macrophages of GBA-PD (L444P/N +N370S/N) patients to controls compared to GBA-PD (L444P/N +N370S/N) patients to GBA carriers (L444P/N +N370S/N), and compared to GBA carriers (L444P/N +N370S/N) and controls; B. DEGs determined be Venn diagram in (**B**) and DEGs determined by GO analysis for GBA-PD (L444P/N +N370S/N) patients, GBA carriers (L444P/N +N370S/N), controls. All data are presented as the number of genes with a *p*-value < 0.05 and |FC| more than 1.5. Three Venn diagrams were developed using the library VennDiagram (v.1.6.20) in R (v.4.0.3). (GBA-PD—Parkinson’s disease associated with mutations in the *GBA* gene; GBA carriers—asymptomatic *GBA* mutation carriers; DEGs—differentially expressed genes; GO—gene ontology).

Comparative transcriptome analysis of monocyte-derived macrophages revealed 23 DEGs between GBA-PD patients and GBA carriers, 28 DEGs between GBA-PD patients and controls. Moreover, eight DEGs were found between GBA carriers compared to controls (Figure 4A,B, Tables S5–S7.) The top list also revealed DEGs in GBA-PD patients compared to controls, including the genes, *JUNB*, *NR4A2*, *EGR1*. Significant terms of GO analysis between GBA-PD patients, GBA carriers, and controls are presented in Table S8, and networks are presented in Figure 5A,B. A total of 25 genes were enriched in 17 GO pathways. The altered biological pathways in GBA-PD patients compared to the controls were directly related to the functioning of the immune system, immune response, cytokine metabolism, and the immune response ((GO:0019221) cytokine-mediated signaling pathway, (GO:0006935) chemotaxis, (GO:0002548) monocyte chemotaxis, (GO:1990869) cellular response to chemokine), and apoptosis ((GO:0010941) regulation of cell death, (GO:0010942) positive regulation of cell death). The main of the alerted GO pathways in GBA carriers compared to the controls was the pathway associated with cytokine metabolism ((GO:0071345) cellular response to cytokine stimulus). The Venn diagram demonstrated two genes *HOOK2*, *JUNB* downregulated in GBA-PD patients and GBA carriers compared to controls that can be attributed to the presence of *GBA* mutations. The *HOOK2*, *JUNB* genes were also involved in the enriched pathway (response to cytokines (GO:0034097)) identified by GO analysis in GBA-PD patients compared to controls (Figure 6A). 

### 3.4. Differential Expression of Genes and Pathways in GBA-PD Patients (L444P/N +N370S/N) and GBA Carriers (L444P/N +N370S/N)

Differential expression analysis of monocyte-derived macrophages resulted in 23 DEGs in GBA-PD patients compared to GBA carriers (Figure 4C). GO analysis showed the main altered pathways that are related to immune response ((GO:0042092) type 2 immune response, (GO:0006952) defense response) (Table S8, Figure 5C). The Venn diagram allowed us to reveal seven overlapping DEGs (*DUSP1*, *ALR4C*, *RPL16*, *TPTEP1*, *COLEC12*, *TRIM13*, *BCL6*) among GBA-PD patients when compared with GBA carriers and controls and two genes (*ACOD1*, *IL31RA*) between GBA carriers compared with GBA-PD patients and controls (Figure 6B). The comparison between the list of DEGs from GO analysis and list of DEGs obtained by the Venn diagram revealed two genes (*IL31RA*, *ACOD1*) downregulated in GBA carriers, compared to GBA-PD and controls, and four deregulated genes (two (*DUSP1*, *COLEC12*) downregulated and two (*TRIM13*, *BCL6*) upregulated) in GBA-PD patients, compared to GBA carriers and controls (Figure 6B). 

### 3.5. Searching the Overlapping DEGs between our and Publicly Available Dataset

To identify the similarities between lists of DEGs from our and previously published studies we used Venn diagram. We revealed overlapping DEGs between list of DEGs from our analysis of GBA-PD (L444P/N +N370S/N) and list of DEGs of G2019S LRRK2-PD from study of Infante and colleagues: two genes encoding monocyte attracting chemokines, such as *CCL3L1* gene, in GBA-PD, G2019S LRRK2-PD and PD in comparison with controls, and the *CCL3* gene, when comparing GBA-PD to controls and G2019 LRRK2-PD to PD and also, *JUNB* gene when comparing GBA-PD, GBA-carriers, G2019 LRRK2-PD and G2019 LRRK2-carriers [15]. Dataset of Infante and colleagues’ study is available for download following this link: https://ars.els-cdn.com/content/image/1-s2.0-S0197458015005382-mmc1.doc [15] (accessed on 9 September 2021). 

## 4. Discussion

This is the first whole-transcriptome analysis of monocyte-derived macrophages in GBA-PD patients, GBA carriers, and controls. We intended to cover molecular pathways involved in GBA-PD pathogenesis and study the differences in the transcriptome between *GBA* mutation carriers with and without PD. To date, the last review of genome-wide transcriptomic studies in sporadic PD identified a total of 96 studies during the period between 2004 and 2017: 12 meta-analyses, 21 re-analyses of exiting data, and 63 original studies carried out by means of different genome-wide technologies [30]. Several studies analyzed transcriptomic profiles in the blood, brain tissue, and dopaminergic neurons in autosomal dominant PD associated with mutations in the *LRRK2* gene (LRRK2-PD) (OMIM no.609007) [13,31,32,33], with only one research study conducted with RNA-seq technology [14]. In fact, presently, only one research study has examined the transcriptomic profile in GBA-PD [34] despite the (obvious) actual problems of incomplete penetrance of *GBA* mutations. 

Here, we compared the gene expression profile in monocyte-derived macrophages between L444P/N *GBA* mutation carriers, discordant for clinical manifestation of PD and controls. This mutation is more severe compared to N370S, and characterized by an earlier age of PD onset, as well as motor, psychiatric, cognitive, and olfactory symptoms [6]. It also results in more pronounced alpha-synuclein accumulation in in vitro and in vivo models of PD [35]. According to our previous data, GCase enzyme activity decreases more strongly and the plasma level of oligomeric alpha-synuclein is higher in L444P/N GBA-PD patients compared to N370S/N GBA-PD patients [10]. We revealed 32 DEGs between L444P/N GBA-PD and the controls, 36 between L444P/N GBA-PD and L444P/N GBA carriers and 18 between L444P/N GBA carriers and controls. First, we focused on searching for molecular biomarkers involved in PD pathogenesis among L444P/N *GBA* mutation carriers. We revealed two potential biomarkers for PD in L444P/N *GBA* mutation carriers (downregulation of the *DUSP1* and *ARL4C* gene expression). The *DUSP1* gene encodes the mitogen-activated protein kinase 1 (MKP-1) phosphatase that participates in regulation of apoptosis, endoplasmic reticulum (ER) stress, cell cycle, and autophagy, with the cellular process playing a pivotal role in PD [36]. MKP-1 belongs to the class I classical cysteine-based protein phosphatases (DUSP family) that have the dual ability to dephosphorylate phospho-serine/threonine and phospho-tyrosine residues [37,38]. MKP-1 is expressed during embryonic development in the midbrain, including dopaminergic neurons, as well as in adulthood in substantia nigra (SN) and can act as a neuroprotective agent. *ARL4C*, known also as *ARL7*, participates in cholesterol transport between the perinuclear compartment and the plasma membrane for ABCA1-associated removal and, thus, may be integral to the LXR-dependent efflux pathway [39]. Dysregulation of cholesterol metabolism has been implicated in PD [40]. 

Next, we aimed to find similarities in symptomatic and asymptomatic L444P/N *GBA* mutation carriers that can be attributed to the presence of L444P/N *GBA* mutations. Moreover, all L444P/N *GBA* mutation carriers were characterized by an increased *KIAA0319* expression level. The *KIAA0319* gene was involved in the pathway associated with the axon extension, involved in regeneration (GO:0048677). The genetic variants of *KIAA0319* were found to be associated with dyslexia [41,42].

Additionally, the transcriptomic analysis was conducted for both L444P and N370S *GBA* mutations. We revealed 28 DEGs between GBA-PD and controls, 23 between GBA-PD and GBA carriers, and 8 between GBA carriers and controls. We suggested that four genes, *DUSP1*, *COLEC12*, *TRIM13*, *BCL6*, deregulated in GBA-PD patients, might be potential candidates for PD biomarkers among *GBA* mutations carriers. Downregulated expression of *DUSP1* and *COLEC12* genes and upregulated expression of the *TRIM13* and *BCL6* genes were found in GBA-PD patients compared to both GBA carriers and controls. *DUSP1* and *TRIM13* are involved in initiation of autophagy and in the ubiquitin-proteasome pathway of protein degradation during ER stress that may play a critical role in alpha-synuclein degradation. It has been shown that repression of endogenous *TRIM13* inhibits autophagy induced by ER stress [43]. Family DUSPs have many substrates and modulate diverse neural functions, such as neurogenesis, differentiation, and apoptosis. DUSP1 critically contributes to the resolution of acute inflammatory responses of macrophages and mediates protective glucocorticoids effects, which potently inhibit pro-inflammatory responses, and are widely used for the treatment of inflammatory diseases [44]. We revealed decreased expression level of the *DUSP1* gene in GBA-PD patients compared to GBA carriers and controls. Thus, a decreased expression level of the DUSP1 gene may lead to impairment of macrophage’s inflammatory response and, therefore, contribute increasing inflammation levels. TRIM13 is a negative regulator of MDA5-mediated type I interferon (IFN) production and may impact RIG-I-mediated type I IFN production. Proper regulation of the type I IFN response contributes to maintaining immune homeostasis [45]. Since macrophages are vital to immune response, dysregulation of the TRIM13 gene may lead to disturbance of immune homeostasis and levels of cytokines, which act as important mediators of the immune system. The *COLEC12* gene, known also as *SCARA4*, *SRCLI*, *SRCLII*, *CL-P1*, is implicated in innate immune responses [46], and is associated with lipid metabolism and phagosome formation. In particularly, *COLEC12* protein functions as a receptor for the detection, uptake, and degradation of oxidized modified low-density lipoproteins by vascular endothelial cells [47]. *BCL6* is a critical marker in cell apoptosis and contributed to the inflammation activation of macrophages [48]. Previous studies on mouse and human macrophages showed that COLEC12 is a novel receptor involved in myelin uptake by phagocytes and may play a role in active multiple sclerosis, which is a chronic, inflammatory, neurodegenerative disease [49]. Considering its role in the uptake of myelin, COLEC12 likely plays an important role in the pathophysiology of neurodegenerative disease, but as an uptake of myelin leads to both demyelination and central nervous system repair, depending on whether it concerns intact myelin or myelin debris, COLEC12-mediated myelin uptake can be beneficial or detrimental. BCL6 is a critical marker in cell apoptosis and contributes to the inflammation activation of macrophages. BCL6 overexpression was found to inhibit the increase in reactive oxygen species ROS. Mitochondrial functions lead to exacerbation of ROS generation and susceptibility to oxidative stress involved in PD pathogenesis [50].

Next, we found similarities in symptomatic and asymptomatic *GBA* mutation carriers that consisted of the decreased *JUNB* and *HOOK2* gene expression in both GBA-PD patients and GBA carriers compared to controls. *HOOK2* encodes the Hook2 protein that belongs to a family of cytoplasmic linker proteins. Hook2 is implicated in the formation of aggresomes, vesicle trafficking, and fusion, particularly in degradation of neuronal tau aggregates in Alzheimer’s disease (AD) [51,52,53]. 

Comparing the transcriptomes between the GBA-PD independent of the type of mutation (L444P, N370S) as well as in L444P/N GBA-PD revealed three genes (*JUNB*, *EGR1*, *NR4A2*) encoding transcriptional regulators involved in the maintenance of dopaminergic neuron function, neuronal differentiation, and neurogenesis from the top of the DEGs list. It is worth noting that a previous transcriptomic analysis conducted for the blood and brain for sporadic PD revealed an alteration in the pathways that include dopamine metabolism, mitochondrial function, oxidative stress, protein degradation, neuroinflammation, vesicular transport, and synaptic transmission [30]. Our data support the statement that neurodegenerative mechanisms could be detectable from a peripheral tissue. *JUNB*, *EGR1*, *NR4A2* belong to immediate-early genes (IEGs) and encode the transcription factors, JunB, Egr-1, NR4A2, respectively [54,55,56,57]. These factors are activated in respond to a variety of cellular stimuli and control specific neuronal functions, including neuronal activity. Both JunB and Egr-1 are key mediators of apoptosis and the inflammatory response [58,59]. It is interesting to note that *JUNB* overexpression protections against cell death of nigral neurons [60]. Furthermore, JunB modulates expression of canonical markers of alternative activation in macrophages [61]. The latest study demonstrated that a large share of EGR1 target regions in macrophages are enhancers associated to the inflammatory response [59]. Egr1 inhibits pro-inflammatory gene expression in macrophages [59]. Egr-1 activation promotes neuroinflammation and dopaminergic neurodegeneration in an experimental model of PD [62]. *NR4A2* (Nurr1) is critical in the development and maintenance of the dopaminergic neurons. It coordinates several key proteins, including tyrosine hydroxylase (TH), dopamine transporter (DAT), and vesicular monoamine transporter (*SCL18A2*/*VMAT*) [63]. Previous studies demonstrated an association between *NR4A2* polymorphisms with PD [64,65,66] and showed that sporadic PD patients is characterized by decreased *NR4A2* gene expression in PBMCs [67]. Nurr1 also appears to restrain inflammatory processes by polarizing macrophages to the M2 type [68]. Thus, the role of these genes in neuroimmune interaction could not be excluded as monocytes; macrophages may migrate across the blood–brain barrier and induce the neuroinflammatory processes in the brain and, therefore, contribute to brain pathology, such as neurodegeneration [69]. 

According to the Human Protein Atlas (https://www.proteinatlas.org (accessed on 9 September 2021)), the top of DEGs in GBA-PD patients compared to controls, *JUNB, EGR1*, *NR4A2*, and potential biomarkers of GBA-PD (*DUSP1, COLEC12, TRIM13, BCL6*, *ARL4C)* express not only in the blood, but in brain tissues. 

GO enrichment analysis revealed several altered pathways in GBA-PD patients independent of the type of mutation in the *GBA* genes (L444P, N370S) generally related to the immune system. Growing evidence suggests that neuroinflammation may contribute to the development of Parkinson’s disease and elevated levels of inflammation-related mediators in the brain and cerebrospinal fluid. Many studies focused on peripheral inflammatory processes have found a significant association between immune markers and disease severity. We should note that, previously, we (and others) demonstrated elevation of proinflammatory cytokine secretion in plasma of GBA-PD patients compared to sporadic PD patients and controls [70,71]. 

Presently, only one paper performed transcriptomic analysis for PD patients baring *GBA* mutations. The study was fulfilled on iPSC-derived dopamine neurons from three GBA-PD patients with the N370S *GBA* variant [34]. Single-cell profiling demonstrated disease relevant pathways, even in the carriers of the same mutation. Thus, in one initially diagnosed as a patient with PD, the patient’s cellular profile prompted a clinical reassessment, leading to a revised diagnosis of progressive supranuclear palsy (PSP). Nevertheless, on iPSC-derived dopamine neurons from two other patients with N370S GBA-PD, the authors found 60 deregulated genes that included downregulated genes implicated in neuronal function, and upregulated genes involved in zinc ion transport [33]. Similar to Lang and colleagues, we also found upregulation of genes, *MT1F*, *MT1L*, *MT1M*, *MT1X*, and *SLC39A8*, involved in the zinc metabolism pathway in GBA-PD patients, compared to GBA carriers. Alterations of zinc homeostasis have long been implicated in PD. Zn2+, besides its role in multiple cellular functions, also acts as a synaptic transmitter in the brain. Recent meta-analysis studies, though, point to lower zinc levels in serum and plasma and CSF of PD patients compared to healthy controls. The association between deregulated levels of circulating zinc and PD has been explained by its antioxidant role since this trace element is essential for a variety of enzymes and proteins (superoxide dismutase oxidative, metallothioneins, and interleukins) involved in oxidative stress [72]. Moreover, dysregulated zinc homeostasis zinc plays a critical role in the innate immune system, especially for maintaining the function of macrophages due to participation in impairment phagocytosis and an abnormal inflammatory response [73]. The following ingenuity pathway analysis conducted by Lang and colleagues showed that, among 60 deregulated genes in GBA-PD, eight (*PRKCB*, *RTN1*, *ATP1A3*, *TSPAN7*, *NTM*, *L1CAM*, *BDNF*, *SLC2A1*) are regulated with histone deacetylase 4 (HDAC4). In our study, both GBA-PD and L444P/N GBA-PD patients demonstrated decreased expression of the *DUSP1* gene involved in ER stress, implicated previously in PD pathogenesis, particularly GBA-PD [74]. Notably, Lang and colleagues found downregulation of another gene from the same MKP family—the *DUSP4* gene encoding MKP-2 that is closely related with *DUSP1*/MPK-1 [34,38]. There are currently few studies assessing the role of the DUSP genes in PD. However, one study reported decreased *DUSP1* mRNA expression in the brain tissue in an idiopathic PD patient [37]. *DUSP1* overexpression protects dopaminergic neurons against neurotoxicity induced with 6-hydroxydopamine in vitro [75]. Strategies aimed at increasing the expression of DUSP1 have been discussed as potential therapeutic approaches for PD [37]. Taken together, our results highlight the potential important role of the DUSP family in the pathogenesis of GBA-PD. To summarize our results with the study by Lang and colleagues, we could make a conclusion about the involvement of the downregulation of genes related to neuronal functions and upregulation of pathways related to immune response and zinc ion homeostasis in GBA-PD pathogenesis. 

It is interesting to note that, oppositely, regarding the number of DEGs attributed to a presence of *GBA* mutations revealed in our preset study, RNA-seq conducted in *LRRK2* G2019S mutation carriers suggested that G2019S mutation in the *LRRK2* gene markedly altered blood transcriptome in comparison with sporadic PD [14]. Infante and colleagues found 174 genes with significant differential expression in the blood between LRRK2-PD patients with G2019S mutation and asymptomatic carriers and 1139 DEGs between asymptomatic carriers of G2019S *LRRK2* mutation and controls [14]. These data allow us to suggest that the *GBA* mutation had less influence on the transcriptome profile in comparison with *LRRK2* mutations. We compared our gene set with the gene set presented in the study by Infante and colleagues, and revealed overlap genes, encoding monocyte-attracting chemokines, such as *CCL3L1* gene, when comparing GBA-PD, G2019S LRRK2-PD, and PD to controls, and the *CCL3* gene, when comparing GBA-PD to controls and G2019 LRRK2-PD to G2019S LRRK2-carriers. That observation supports the hypothesis involving the role of immune response in PD pathogenesis [76]. It is also important to mention that the difference between the amount of the revealed differentially expressed genes in our study compared to the study by Infante and colleagues can be explained due to the fact that their study conducted whole-blood transcriptomic analysis. We could not exclude the possibility that such discrepancies are attributed to monotype cell populations used in the present study for transcriptomic analysis. 

The current study has some limitations. The small size of the studied groups may influence the outcome of differential expression analysis for genes with small differences in expression levels, eliminating nonspecific gene expression differences. Moreover, the influence of L-DOPA treatment in GBA-PD patients on the gene expression level cannot be ruled out. In addition, we could not exclude PD manifestation among GBA carriers later in their lives, as only 10% of carriers of mutations in the *GBA* gene develop PD at the age of 60, 16% at the age of 70, and 19% at the age of 80 [77]. It is interesting to note that Lang et al. demonstrated that the genome profile in sporadic PD could—in some cases—be similar to GBA-PD, suggesting that findings from GBA-PD could be extrapolated to a subset of sporadic PD patients [34]. A further limitation of our study is the absence of PD patients without *GBA* mutations. 

## 5. Conclusions

In conclusion, this study provides new insights into the global transcriptome in GBA-PD and asymptomatic *GBA* mutation carriers. Potential involvement of genes of neuronal functions, inflammation, and zinc metabolism in the pathogenesis of GBA-PD was shown. Alteration expression of *DUSP1* may be considered a potential biomarker of PD among *GBA* mutations carriers. This knowledge could assist in answering the fundamental question about potential triggers, which is important for future studies devoted toward determining the pathogenesis of PD among *GBA* mutations carriers. 

## Figures and Tables

**Table 1:** Demographic characteristics of the compared groups.

Groups	Age at Exam, Mean ± SD, Years	Age at Onset, Mean ± SD, Years	Gender (Male:Female)	Mutations in the *GBA* Gene
GBA-PD, *N* = 5	53.5 ± 8.73	49.0 ± 10.89	3:2	4 L444P/N 1 N370S/N
GBA-carriers, *N* = 4	54.9 ± 8.9	-	2:2	3 L444P/N 1 N370S/N
Controls, *N* = 4	54.4 ± 9.5	-	2:2	-

GBA-PD—Parkinson’s disease associated with mutations in the *GBA* gene; GBA-carriers—asymptomatic *GBA* mutation carriers; SD—standard deviation.

**Table 2:** Functional clusters selected according to the results of the GO analysis between L444P/N GBA-PD patients, L444P/N GBA carriers, and controls.

(GO ID] GO Terms	p_adjusted_	DEGs
**L444P/N GBA-PD vs. Controls**		
(GO:0097529) myeloid leukocyte migration	6.93 × 10^−9^	*CCL3, CCL3L1, CCL4, CXCL2, CXCL5, DUSP1, IL6, PPBP*
(GO:0002548) monocyte chemotaxis	6.93 × 10^−9^	*CCL3, CCL3L1, CCL4, DUSP1, IL6*
(GO:1990869) cellular response to chemokine	6.93 × 10^−9^	*CCL3, CCL3L1, CCL4, CXCL2, CXCL5, DUSP1, PPBP*
(GO:0030593) neutrophil chemotaxis	6.93 × 10^−9^	*CCL3, CCL3L1, CCL4, CXCL2, CXCL5, PPBP*
**L444P/N GBA-PD vs. L444P/N GBA carriers**		
(GO:0006882) cellular zinc ion homeostasis	8.09 × 10^−9^	*MT1F, MT1L, MT1M, MT1X, SLC39A8*
(GO:0010273) detoxification of copper ion	9.81 × 10^−7^	*MT1F, MT1L, MT1M, MT1X*
(GO:0071276) cellular response to cadmium ion	9.81 × 10^−7^	*MT1F, MT1L, MT1M, MT1X*
(GO:0071280) cellular response to copper ion	9.81 × 10^−7^	*MT1F, MT1L, MT1M, MT1X*
(GO:0071294) cellular response to zinc ion	9.81 × 10^−7^	*MT1F, MT1L, MT1M, MT1X*
**L444P/N GBA carriers vs. controls**		
(GO:0048731) system development	0.001035	*ACOD1, IL31RA, KIAA0319, OSCAR, TBX3*
(GO:0002520) immune system development	0.001035	*ACOD1, IL31RA, OSCAR*
(GO:0002573) myeloid leukocyte differentiation	0.001011	*IL31RA, OSCAR*
(GO:0048692) negative regulation of axon extension involved in regeneration	0.000615	*KIAA0319*
(GO:0048677) axon extension involved in regeneration	0.000615	*KIAA0319*

GBA-PD—Parkinson’s disease associated with mutations in the *GBA* gene; GBA carriers—asymptomatic *GBA* mutation carriers; DEGs—differentially expressed genes; GO—gene ontology.

## Data Availability

The data discussed in this publication have been deposited in NCBI’s Gene Expression Omnibus (Edgar et al., 2002) and are accessible through GEO Series accession number GSE184956 (https://www.ncbi.nlm.nih.gov/geo/query/acc.cgi?acc=GSE184956 (accessed on 9 September 2021)) and at ArrayExpress database at EMBL-EBI (www.ebi.ac.uk/arrayexpress/ (accessed on 9 September 2021)) under accession number E-MTAB-11029 or https://www.ebi.ac.uk/arrayexpress/arrays/E-MTAB-11029 (accessed on 9 September 2021) for array design “E-MTAB-11029”.

## Supplementary Materials

The following are available online at https://www.mdpi.com/article/10.3390/genes12101545/s1, Table S1: Post-trimming and mapping results to reference genome for all groups, Table S2: Differentially expressed genes from L444P/N GBA-PD patients compared to controls in monocyte-derived macrophages, Table S3: Differentially expressed genes from L444P/N GBA-carriers compared to controls in monocyte-derived macrophages, Table S4: Differentially expressed genes from L444P/N GBA-PD patients compared to L444P/N GBA-carriers in monocyte-derived macrophages, Table S5: Differentially expressed genes from GBA-PD (L444P/N+N370S/N) patients compared to controls in monocyte-derived macrophages, Table S6: Differentially expressed genes from GBA-carriers (L444P/N+N370S/N) compared to controls in monocyte-derived macrophages, Table S7: Differentially expressed genes from GBA-PD (L444P/N+N370S/N) patients compared to GBA-carriers (L444P/N+N370S/N) in monocyte-derived macrophages, Table S8: Functional clusters selected according to the results of GO analysis between GBA-PD (L444P/N+N370S/N) patients, GBA-carriers (L444P/N+N370S/N) and controls.
