# Supplementary material for: Comparative Transcriptome Analysis in Monocyte-Derived Macrophages of Asymptomatic GBA Mutation Carriers and Patients with GBA-Associated Parkinson’s Disease
Source: Genes (Basel). 2021 Sep 29;12(10):1545. doi: 10.3390/genes12101545 (PMC8535749; doi:10.3390/genes12101545)
Supplement: Supplementary file 1 [file genes-12-01545-s001.zip › genes-1394982-supplementary.pdf]

Table S 1. Post-trimming and mapping results to reference genome for all groups

Total reads	Mapping reads	Mapping >1 time	Mapping 1 time	Mapping 0 time
**GBA-PD**				
12245034	94.47%	1390912	10177533	676589
12534145	94.15%	1522375	10278974	732796
13364587	96.02%	1616992	11215592	532003
14588079	96.17%	1520881	12507829	559369
22038017	95.96%	2889324	18258227	890466
**GBA-carriers**				
16815993	94.19%	1868206	13970248	977539
20016766	96.44%	2595136	16708395	713235
11328352	95.71%	1415542	9427273	485537
15042608	95.42%	2338602	12014382	689624
**Controls**				
13186256	94.77%	1922697	10573358	690201
12888917	94.42%	1349391	10820389	719137
29105091	96.34%	2980649	25060066	1064376
13276643	95.33%	1916805	10740334	619504

GBA-PD - Parkinson's disease associated with mutations in the GBA gene; GBA-carriers - asymptomatic GBA mutation carriers

Table S 2. Differentially expressed genes from L444P/N GBA-PD patients compared to controls in monocyte-derived macrophages

DEGs	padj	log2FoldChange
JUNB	1.56×10^{-8}	−1.273329
HOOK2	2.39×10^{-7}	−1.109320
EGR1	3.17×10^{-6}	−4.187828
DUSP1	3.95×10^{-6}	−3.25103
IL6	3.95×10^{-6}	−3.4155
ARL4C	5.06×10^{-6}	−2.147519
NR4A2	2.94×10^{-4}	−3.001167
CCL3L1	4.026×10^{-4}	−5.2936939
PPBP	0.00123	7.01866736
RPL18	0.00149	−0.724112
IER2	0.003892	−2.148075
TRBV5−1	0.00389	−2.648457

CXCL2	0.00412	−2.436204
MAL	0.00412	−3.526525
MMP7	0.0052	3.5695832
CCL3	0.0052	−2.8581180
DUSP2	0.00665	−2.0777352
ATP2B1−AS1	0.0087	−2.08186683
CCL4	0.0095	−3.10118915
CCL4L2	0.0111	−4.86057595
MIR34AHG	0.0123	1.293966592
KIAA0319	0.0173	2.7304552
JUN	0.0202	−1.8078662
TPTEP1	0.0246	2.2196251
ZFP36	0.03487	−1.5978346
IER3	0.0348	−1.26935
TTLL4	0.0412	0.89197963
COLEC12	0.0412	−2.26691
CXCL5	0.041	2.862093
ENPP2	0.0427	−3.299636
ABHD2	0.0427133	0.90893
HSPA1B	0.042713	0.996150

GBA-PD - Parkinson's disease associated with mutations in the GBA gene; GBA-carriers - asymptomatic GBA mutation carriers; DEGs - differentially expressed genes

Table S 3. Differentially expressed genes from L444P/N GBA-carriers compared to controls in monocyte-derived macrophages

DEGs	padj	log2FoldChange
IL31RA	9.87×10^{-18}	−19.44049
ACOD1	4.78×10^{-14}	−18.67938
PRSS22	4.94×10^{-6}	−19.411937
LINC01891	4.055×10^{-4}	−18.4905145
ATP1B4	4.70×10^{-4}	−21.902312
TBX3	4.70×10^{-4}	−21.694237

VAT1L	0.00100337	−16.8595347
NXPH3	0.0013055	−20.8813694
MT1M	0.0014474	−7.5301355
SHC4	0.002294	−20.309837
HLA−J	0.002965	3.1020326
SPRY4	0.004660	−4.39827985
KCNMB1	0.008565	−2.8714975
JAKMIP2	0.0094078	1.4094559
OSCAR	0.0098210	−1.30014117
KIAA0319	0.016259	3.2499566
FGL2	0.0314813	−2.086525

GBA-PD - Parkinson's disease associated with mutations in the GBA gene; GBA-carriers - asymptomatic GBA mutation carriers; DEGs - differentially expressed genes

Table S 4. Differentially expressed genes from L444P/N GBA-PD patients compared to GBA-carriers in monocyte−derived macrophages

DEGs	padj	log2FoldChange
IL31RA	3.42×10^{-28}	22.81419
ACOD1	4.92×10^{-24}	22.49465
VAT1L	1.62×10^{-7}	21.26457
ATP1B4	1.77×10^{-7}	26.877062
PRSS22	3.74×10^{-7}	20.0742
MT1M	5.64×10^{-7}	9.60953
SHC4	1.52×10^{-5}	23.66668
UTS2R	4.197×10^{-5}	18.4369
NXPH3	4.62×10^{-5}	22.674242
KCNMB1	7.34 × 10−*5*	3.408771
FGD5	7.34×10^{-5}	3.005397
TBX3	1.57×10^{-4}	21.40274
MT1F	1.87×10^{-4}	3.56241
HSPH1	2.75×10^{-4}	0.75877
SPRY4	3.032×10^{-4}	4.830094
KCNJ2	4.24×10^{-4}	2.810203

MT1L	5.049×10^{-4}	6.71591
LINC01891	0.0010437551433765133	16.980
ARL4C	0.0011152363202132764	−1.954802
DUSP1	0.002709	−2.793141
OSCAR	0.004080	1.2904701
TGM2	0.007633753	2.083364
SRSF5	0.008446	−0.616122
UNC5B	0.010984	2.2372
BATF2	0.0218465	2.8215
MT1X	0.027678	3.16545
SLC39A8	0.0284	2.780923830
ZNF704	0.03119	−2.44145
CALHM6	0.03119	4.1001527
STON2	0.0320245	−5.194386
SERPINH1	0.035644	0.92875318
LIPM	0.036739	9.097984
RNF144B	0.03789	1.6824349
NT5C3B	0.0378908	−1.271271
SNHG3	0.04101556	−0.962452

GBA-PD - Parkinson's disease associated with mutations in the GBA gene; GBA-carriers - asymptomatic GBA mutation carriers; DEGs - differentially expressed genes

Table S 5. Differentially expressed genes from GBA-PD (L444P/N+N370S/N) patients compared to controls in monocyte-derived macrophages

DEGs	padj	log2FoldChange
JUNB	2.37×10^{-12}	−1.253670176
HOOK2	2.88×10^{-9}	−1.071183696
EGR1	4.68×10^{-6}	−4.119209619
ARL4C	1.07×10^{-5}	−2.025843567
NR4A2	3.91×10^{-5}	−2.986036049
IL6	6.95×10^{-5}	−3.866493754
DUSP1	0.000139485	−3.369818756

MMP7	0.000489067	3.915533725
CCL3L1	0.000770649	−5.74901087
PPBP	0.000821926	6.654290865
TRBV5−1	0.001747096	−2.531049969
RPL18	0.001949174	−0.703837045
IER2	0.00485697	−2.103641578
TPTEP1	0.005948528	2.203616802
ZFP36	0.007195823	−1.668224122
TRIM13	0.00850527	0.725108438
ENPP2	0.009314153	−3.404150223
VMP1	0.012172779	0.624239627
CCL4L2	0.01305784	−5.208863091
BCL6	0.0210965	0.756069963
CCL3	0.021273736	−2.691408178
COLEC12	0.02135696	−2.182528256
MAL	0.02135696	−3.03596868
CXCL2	0.027199467	−2.713284496
SLC4A8	0.03342678	1.181900735
CYP4V2	0.035836308	0.789161862
SKIL	0.039183179	0.637145847
DOCK5	0.045685169	1.06312728

Table S 6. Differentially expressed genes from GBA-carriers (L444P/N+N370S/N) compared to controls in monocyte−derived macrophages

DEGs	padj	log2FoldChange
IL31RA	2.36×10^{-18}	−17.683476
ACOD1	2.88×10^{-18}	−18.210591
NXPH3	$1.77 \times 10–^{16}$	−29.984238
LINC01891	3.37×10^{-16}	−29.999458
VAT1L	0.00032722	−14.087655
ATP5PDP3	0.00045205	18.7100791

JUNB	0.00046733	−0.8331051
HOOK2	0.00632252	−0.7218695

GBA-PD - Parkinson's disease associated with mutations in the GBA gene; GBA-carriers - asymptomatic GBA mutation carriers; DEGs - differentially expressed genes

Table S 7. Differentially expressed genes from GBA-PD (L444P/N+N370S/N) patients compared to GBA-carriers (L444P/N+N370S/N) in monocyte-derived macrophages

DEGs	padj	log2FoldChange
ACOD1	1.43×10^{-30}	21.1020452
IL31RA	2.00×10^{-28}	19.8053162
IFNB1	1.40×10^{-12}	−30.807576
ARL4C	0.00087384	−1.6917533
TGM2	0.00087384	2.14653337
BCL6	0.00664798	0.82786078
DEPP1	0.00664798	1.83107981
RPL18	0.00795303	−0.6557988
STON2	0.00996956	−4.4615928
KCNE1	0.01102564	2.05763841
YPEL4	0.01374026	1.92204556
LOC100507642	0.01643034	1.07536814
TPTEP1	0.02159264	2.03424689
KLF2	0.02159264	−1.8023782
CXCL5	0.03068743	2.88289408
DUSP1	0.03965958	−2.4511962
COLEC12	0.03965958	−2.0787807
CHST15	0.03965958	0.77291653
TRIM13	0.03965958	0.65318516
BTBD19	0.03965958	1.89239807
HDGFL3	0.04210421	1.05565628
RPL6P19	0.04210421	−1.2254401
ABCC6	0.04819162	1.71221141

GBA-PD - Parkinson's disease associated with mutations in the GBA gene; GBA-carriers - asymptomatic GBA mutation carriers; DEGs - differentially expressed genes

Table S 8. Functional clusters selected according to the results of GO analysis between GBA-PD (L444P/N+N370S/N) patients. GBA-carriers (L444P/N+N370S/N) and controls

[GO ID] GO term	$p_{adjusted}$	DEGs
**GBA-PD (L444P/N+N370S/N) vs controls**		
[GO:0010941] regulation of cell death	0.039	*BCL6. CCL3. DUSP1. EGR1. IL6. MAL. NR4A2. SKIL. TRIM13. ZFP36*
[GO:0010942] positive regulation of cell death	0.0023	*BCL6. CCL3. DUSP1. EGR1. IL6. MAL. SKIL. TRIM13*
[GO:0006935] chemotaxis	0.0015	*CCL3. CCL3L1. CCL4L1. CXCL2. DUSP1. ENPP2. IL6. PPBP*
[GO:0006954] inflammatory response	0.0041	*BCL6. CCL3. CCL3L1. CCL4L1. CXCL2. IL6. PPBP. ZFP36*
[GO:0034097] response to cytokine	2.11×10^{-6}	*BCL6. CCL3. CCL3L1. CCL4L1. CXCL2. DUSP1. EGR1. HOOK2. IL6. JUNB. PPBP. SKIL. ZFP36*
[GO:0030595] leukocyte chemotaxis	1.77×10^{-5}	*CCL3. CCL3L1. CCL4L1. CXCL2. DUSP1. IL6. PPBP*
[GO:0097529] myeloid leukocyte migration	1.31×10^{-5}	*CCL3. CCL3L1. CCL4L1. CXCL2. DUSP1. IL6. PPBP*
[GO:0019221] cytokine−mediated signaling pathway	0.00054	*BCL6. CCL3. CCL3L1. CCL4L1. CXCL2. EGR1. IL6. JUNB. PPBP*
[GO:0005125] cytokine activity	0.00046	*CCL3. CCL3L1. CCL4L1. CXCL2. IL6. PPBP*
[GO:0002548] monocyte chemotaxis	1.67×10^{-5}	*CCL3. CCL3L1. CCL4L1. DUSP1. IL6*
[GO:0048247] lymphocyte chemotaxis	0.033	*CCL3. CCL3L1. CCL4L1*
[GO:1990869] cellular response to chemokine	1.85×10^{-6}	*CCL3. CCL3L1. CCL4L1. CXCL2. DUSP1. PPBP*
**GBA-PD (L444P/N+N370S/N) vs GBA-carriers (L444P/N+N370S/N)**		
[GO:0042092] type 2 immune response	0.00093	*BCL6. IFNB1. IL31RA*
[GO:0002520] immune system development	0.047	*ACOD1. BCL6. IFNB1. IL31RA. KLF2. STON2*
[GO:0002367] cytokine production involved in immune response	0.025	*BCL6. IFNB1. IL31RA*
[GO:0001818] negative regulation of cytokine production	0.019	*ACOD1. BCL6. IFNB1. KLF2*
[GO:0071345] cellular response to cytokine stimulus	0.0095	*ACOD1. BCL6. CXCL5. DUSP1. IFNB1. IL31RA. KLF2*
[GO:0006952] defense response	0.022	*ACOD1. BCL6. COLEC12. CXCL5. IFNB1. IL31RA. TGM2. TRIM13*
**GBA-carriers (L444P/N+N370S/N) vs controls**		
[GO:0071345] cellular response to cytokine stimulus	0.0032	*ACOD1. HOOK2. IL31RA. JUNB*

GBA-PD - Parkinson’s disease associated with mutations in the GBA gene; GBA-carriers - asymptomatic GBA mutation carriers; DEGs - differentially expressed genes
